# Manufacturing process of short carbon fiber reinforced Al matrix with preformless and their properties

**DOI:** 10.1038/s41598-021-02915-7

**Published:** 2021-12-03

**Authors:** Yongbum Choi, Xuan Meng, Zhefeng Xu

**Affiliations:** 1 Mechanical Program, Graduate School of Advanced Science and Engineering, Hiroshima University, 1-4-1 Kagamiyama, Higashi-Hiroshimasi, Hiroshima, 739-8527 Japan; 2 Department of Mechanical Science and Engineering, Graduate School of Engineering, Hiroshima University, 1-4-1 Kagamiyama, Higashi-Hiroshimasi, Hiroshima, 739-8527 Japan; 3 State Key Laboratory of Metastable Materials Science and Technology, Yanshan University, Hebei Street 438#, Qinhuangdao, 066004 China

**Keywords:** Structural materials, Composites, Mechanical properties

## Abstract

The conventional manufacturing process of fiber-reinforced metal matrix composites via liquid infiltration processes, preform manufacturing using inorganic binders is essential. However, the procedure involves binder sintering, which requires high energy and long operating times. A new fabrication process without preform manufacturing is proposed to fabricate short carbon fiber (SCF)-reinforced aluminum matrix composites using a low-pressure infiltration method. To improve the wettability between fiber and matrix, fibers were plated copper using an electroless plating process. The low-pressure infiltration method with preformless succeeded in manufacturing a composite with a volume fraction of about 30% of carbon fibers.The fiber orientation of the composite material manufactured without preform and the fiber orientation of the composite material manufactured using an inorganic binder was almost the same. The manufactured composites with preformless have high hardness and high thermal conductivity.

## Introduction

Carbon fiber (CF) is one of the most appealing reinforcements and possesses low density, high tensile strength, and high thermal conductivity (TC)^1^. CF- reinforced aluminum composites combine the properties of CF and Al, exhibiting low density and excellent mechanical and thermal/electrical properties^2^, and are expected to be utilized as heat sink materials. The conventional infiltration processes for fabricating SCF reinforced Al matrix composites generally use inorganic binders such as SiO_2_^3^, Al_2_O_3_^4^, or TiO_2_^5^ for the SCF preform manufacturing. However, preform manufacturing with an inorganic binder is complicated, and the preform needs high temperatures and more time for binder sintering. Therefore, a novel fabrication process that is cost-effective with high production efficiency without requiring preform manufacturing is needed for manufacturing SCF/Al composites.

In the liquid infiltration process for fabricating CF-reinforced Al matrix composites, problems such as poor wettability between CF and Al matrix, and the high reactivity of CFs with Al and its alloys, forming undesirable interfacial reaction products (e.g., Al_4_C_3_), limit the applications of CF reinforced Al matrix composites^6^. To solve these, surface modification of CF is proposed as the most effective way to improve wettability and prevent the formation of interfacial reactions. Copper (Cu) has been proven to be effective as it improves wettability, thermal conductivity, and impact strength^7^. The low-pressure infiltration process (LPI)^8^ has been widely adopted for manufacturing composites due to the advantage of requiring relatively simple facilities and being cost-effective.

The objective of this research is to develop a new process for fabricating SCF reinforced Al matrix composites without preform manufacturing. An electroless Cu plating process was conducted on the SCFs to improve wettability. The most suitable electroless Cu plating conditions and infiltration conditions were analyzed. In addition, the effect of SCF content on Vickers hardness and thermal conductivity of composites without preform manufacturing was also investigated.

## Experimental procedure

**Figure 1:**
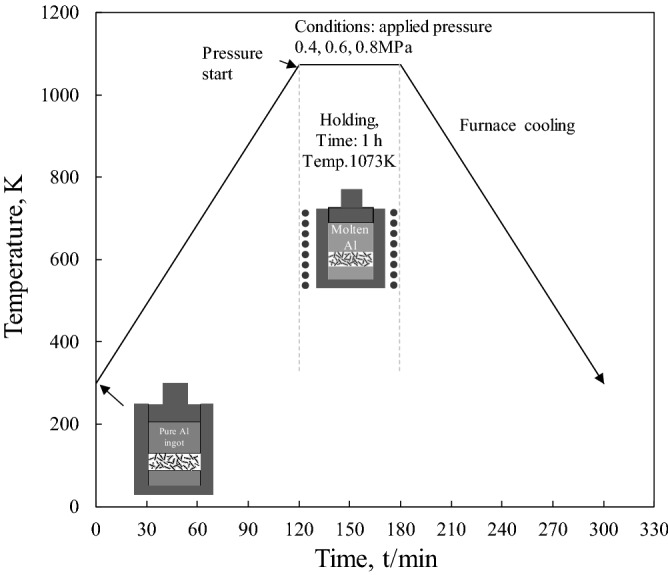
Manufacturing process of short carbon fiber reinforced Al composite by preformless.

A1070 with a purity of 99.7% was used as the matrix in this experiment. Pitch-based SCF (K13D2U, Mitsubishi Plastics, Inc.), with a diameter and aspect ratio of 11 μm and 230, respectively, was used as the reinforcement. To skip the preform manufacturing process, electroless Cu-plating was conducted on SCFs for the 30–120 s. The pH and temperature of the electroless plating bath were 12 and 298 K, respectively. The electroless Cu plating process is summarized in Table 1. Cu-plated SCFs were put into a graphite mold of diameter 10 mm with height adjusted to 10 mm to control the volume fraction of CF, the A1070 ingot was placed on the SCFs and graphite, and a punch was placed on top of the A1070 ingot. The composites were fabricated via the LPI process at 1073 K in an Argon atmosphere by applying 0.4, 0.6, and 0.8 MPa of pressure. The volume fraction of SCFs ranged from 7.1 to 29.1 vol%. Figure 1 shows the Manufacturing process of short carbon fiber reinforced Al composite by preformless. Besides, SCF preform of 10 vol.% reinforced composite fabricated with SiO_2_ binder^9^ was used in the comparison of fiber dispersion in composites. The microstructures of the Cu-plated SCFs and Cu-plated SCF/Al matrix composites without preform manufacturing were observed using a scanning electron microscope (SEM, TOPCON SM-520, Japan). The relative density of each composite material was measured by the Archimedes method.The Vickers hardness test was carried out using a load of 3 kg for 10 s. The thermal conductivity of Cu-plated SCF/A1070 composites without preform manufacturing was measured by laser flash method thermal constants measuring system (TC-9000H, ULVAC-RIKO Inc., Japan) at room temperature. Thermal conductivity was evaluated according to ASTM E 1461-01.

**Table 1:** Solutions used for the different stages in electroless Cu plating.

Stage	Solution
(a) Sensitization 15 min at room temperature	12 g/l SnCl_2_·2H_2_O
	40 ml/l HCl
(b) Activation 15 min at room temperature	0.2 g/l PdCl_2_·2H_2_O
	2.5 ml/l HCl
Metallization	10 g/l CuSO4·5H_2_O
pH 12	45 g/l EDTA
	20 g/l NaCOOH
	16 ml/l HCHO 36%
	NaOH for adjusting pH

## Results and discussions

### Microstructures of Cu-plated SCFs and Cu-plated SCF/Al matrix composites

**Figure 2:**
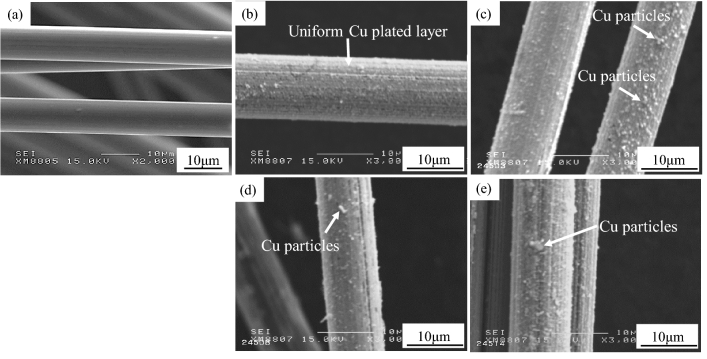
SEM images of (**a**) as-received SCF, (**b**) Cu-plated SCF, 30 s, (**c**) Cu-plated SCF(60 s), (**d**) Cu-plated SCF, 90 s, and (**e**) Cu-plated SCF, 120 s.

Figure 2 shows the SEM images of as-received SCF and Cu-plated SCFs at different plating times. It was observed that the as-received SCFs exhibited a clean and smooth surface, as shown in Fig. 2a. Figure 2b illustrates that a perfect, uniform Cu-plated layer was acquired over the SCFs after plating for 30 s, which could provide uniform wettability and protection. With increasing plating time, the Cu particles attached to the SCF surface lead to an uneven plating layer as shown by the arrows (Fig. 2c–e). Table 2 shows the average Cu plating layer thickness of SCFs and the density of Cu-plated SCFs at different plating times. The Cu plating thickness and the density of Cu plated SCFs increased with increasing plating time. The plating thickness varied from 0.81 to 1.12 µm and the density of Cu-plated SCFs varied from 2.24 to 2.68 kg/m^3^. As the density of liquid aluminum is equal to 2.35 kg/m^3^ at 933 K^10^, the density of SCFs plated for the 30 s was similar to that of molten Al, which contributed to fiber dispersion during infiltration. Therefore, the SCFs plating resulting from 30 s of Cu plating was used for fabricating composites.

**Table 2:** Average Cu-plated thickness and density of Cu-plated SCF by different plating times.

Plating time (s)	Average thickness (µm)	Density of Cu-plated SCF (kg/m^3^)
30	0.81	2.24
60	1.01	2.42
90	1.05	2.50
120	1.12	2.68

**Figure 3:**
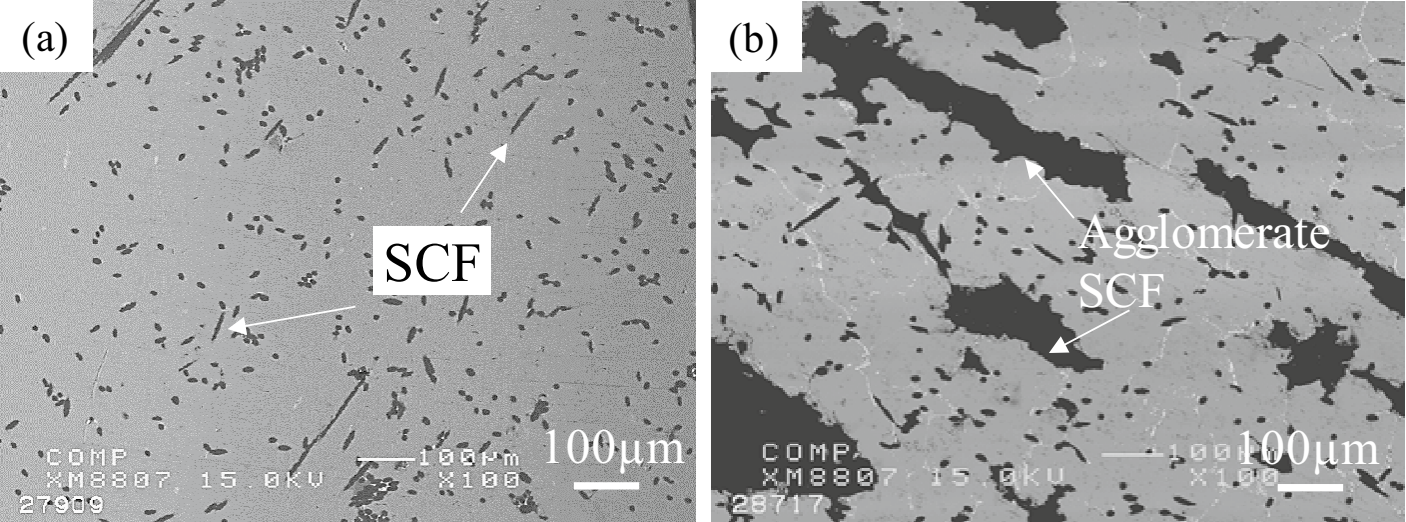
Microstructures of Cu-plated SCF and as received SCF reinforced A1070 composites without preform manufacturing under pressure of 0.8 MPa: (**a**) Cu-plated SCF, (**b**) as-received SCF without Cu plating.

**Figure 4:**
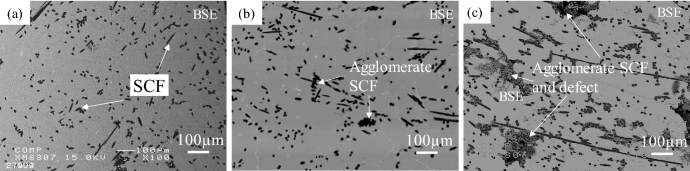
Microstructures of Cu-plated SCF/A1070 composites without preform manufacturing with different applied pressure under containing 7.1 vol% SCF: (**a**) 0.8 MPa, (**b**) 0.6 MPa, and (**c**) 0.4 MPa.

**Figure 5:**
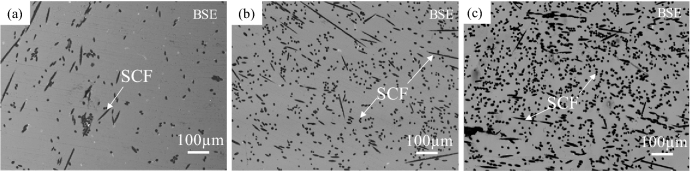
Microstructures of Cu-plated SCF/A1070 composites without preform manufacturing with different volume fraction of SCF under pressure of 0.8 MPa: (**a**) 7.1, (**b**) 14.3, and (**c**) 29.1 vol%.

**Figure 6:**
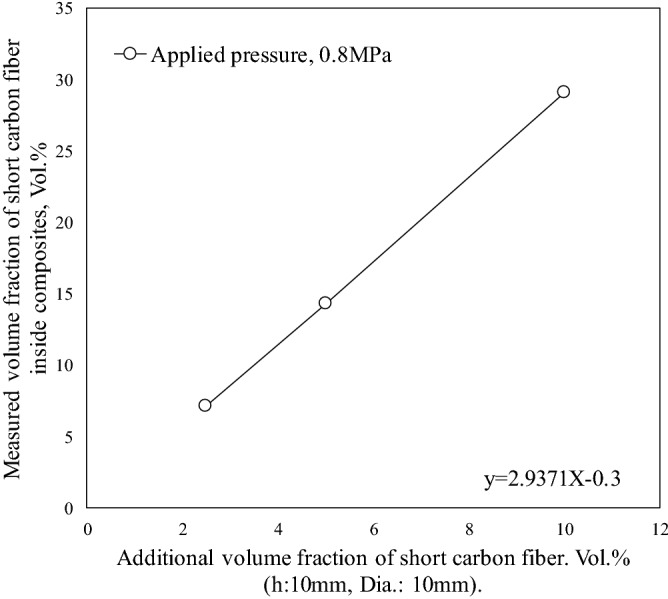
Relationship between additional volume fraction of short carbon fiber and measured volume fraction of short carbon fiber.

**Figure 7:**
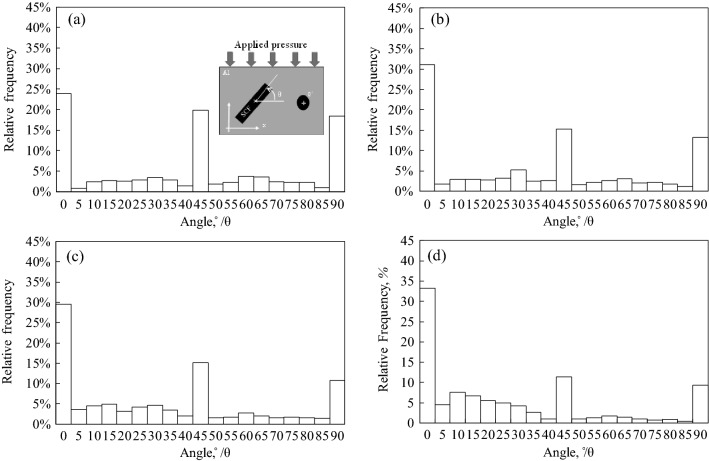
Relative frequency distribution of SCFs in cross-section of composites: (**a**) Cu-plated SCF (7.1 vol%)/Al composite, (**b**) Cu-plated SCF (14.3 vol%)/Al composite, (**c**) Cu-plated SCF (29.1 vol%)/Al composite, and (**d**) SCF prefrom of 10 vol% with SiO_2_ binder /Al composite.

Figure 3 shows the microstructures of Cu-plated SCF (7.1 vol%) and as-received SCF (7.1 vol%) reinforced A1070 composites without preform manufacturing under a pressure of 0.8 MPa. Cu-plated SCFs presented good wettability with the Al matrix and SCFs were randomly dispersed in the composite by dissolving the Cu-plated layer into the matrix, as shown in Fig. 3a. However, in the as-received SCF without Cu plating reinforced A1070 composite, as shown in Fig. 3b, most SCFs were agglomerated in the composite due to not good wettability and poor fiber dispersibility. The agglomeration of the fibers leads to imperfect infiltration defects which decreases the mechanical and thermal properties of composites. Figure 4 shows the microstructures of Cu-plated SCF (7.1 vol%) reinforced A1070 composites without preform manufacturing at different pressures. SCFs exhibited the best random dispersion situation when fabricated under 0.8 MPa, as shown in Fig. 4a. However, SCFs exhibited more agglomeration, which led to larger defects as the fabricated pressure (0.6 and 0.4 MPa), as shown in Fig. 4b and c. In the case of the pressure applied to the molten metal being less than 0.6 MPa, SCFs were unable to be dispersed due to the lack of pressure between the plated SCF and CFs, and the defects between CFs increased. Therefore, the infiltration pressure of 0.8 MPa was the most suitable pressure for fabricating composites with different volume fractions of Cu-plated SCFs. Figure 5 shows the microstructures of Cu-plated SCF/A1070 composites without preform manufacturing with different volume fractions of CF. It is seen that the Al matrix thoroughly penetrated the SCFs, was well bonded to the SCFs, and exhibited good dispersion of CFs. Figure 5a, the SCFs containing 7.1 vol% were more or less uniformly distributed, and that some fiber clusters were present in the composites. As the fiber content increased up to 14.3 vol%, the uniformity of distribution of the SCFs in the composite increased (Fig. 5b). It is difficult to fabricate SCF/Al matrix composite with an SCF content above 20 vol% using the conventional fabrication process with SCF preform with SiO_2_ binder. The Cu-plated SCF/A1070 composites without preform manufacturing containing 29.1 vol% SCFs are shown in Fig. 5c. Most SCFs were uniformly distributed in the Al matrix which proved that Cu-plated SCFs showed better dispersibility. However, uniformity in the distribution of fibers decreased because of the agglomeration of fibers in the composites due to high fiber volume fraction. The relationship between the amount of SCF with height 10 mm and diameter 10 mm and the volume fraction of SCF after the manufacturing composite material is shown in Fig. 6. Compared with the volume fraction of SCF added before the composite material is manufactured, the volume fraction of SCF after the composite material is manufactured is increased. This is due to the shrinkage of SCF at a pressure of 0.8 MPa. Since it is a process that does not manufactured preform process when manufacture composite materials, some fiber shrinkages are generated when pressure is applied. However, the relationship between the amount of SCF added and the volume fraction of SCF in the composite material is established. Relative frequency distribution of SCFs in the Cu-plated SCF/Al matrix composites fabricated without preform manufacturing with different fiber volume fraction and in SCF preform of 10 vol.% reinforced Al matrix composite fabricated with SiO_2_ binder are shown in Fig. 7. In the Cu-plated SCF/Al matrix composites without preform manufacturing in Fig. 7a–c, SCFs were distributed in each direction and mainly oriented at 0°, 45°, and 90°. It is seen that the SCFs exhibited good dispersibility and flowability in composites.

**Figure 8:**
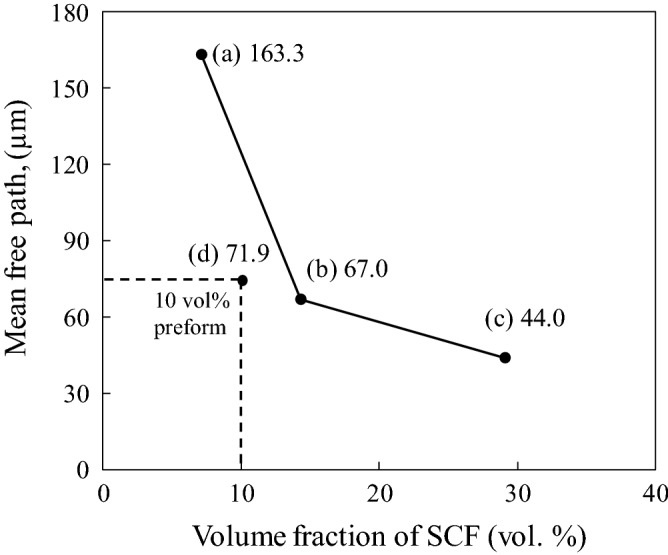
Image mean free path (IMFP) of SCF/Al composites with different volume fractions of short carbon fiber.

On one hand, Cu-plated SCFs possessed a density similar to molten Al which can be flowed with molten Al during the infiltration process. On the other hand, the Cu-plated layer was dissolved into the matrix which obviously improved the wettability between the SCFs and Al matrix, endowing SCFs with better dispersibility. Figure 7d shows the relative frequency distribution of fibers mainly oriented at 90° in composite fabricated with SiO_2_ binder. This is attributed to the SCFs orientation which was perpendicular to the pressing direction during the preform manufacturing process. As a result, most SCFs were perpendicular to the infiltration direction. Figure 8 shows the image mean free path (IMFP) of the Cu-plated SCF/Al matrix composites fabricated without preform manufacturing with different fiber volume fraction and SCF preform reinforced Al matrix composite fabricated with SiO_2_ binder. The Cu-plated SCF/Al matrix composites containing 7.1, 14.3, and 29.1 vol% SCFs showed IMFPs of 163.3, 67.0, and 44.0 µm, respectively, as shown in Fig. 8a–c. The mean free path decreased as the volume fraction of SCFs increased, causing the microstructure of composites to become much finer as the volume fraction of SCFs increased. The introduction of SCFs can prevent grain growth leading to fine grains in composites. As for the SCF preform of 10 vol% reinforced Al matrix composite fabricated with SiO_2_ binder, the mean free path was 71.9 µm, as shown in Fig. 8d. The mean free path of the composite containing 10 vol.% of SCF fabricated preform with SiO_2_ binder was much lower than the predicted value of the composite containing 10 vol% SCFs without preform process. It was proven that the new fabrication process without preform manufacturing enables the production of composites with refined microstructures and higher reinforcement volume fractions.

### Vickers hardness and thermal conductivity of each composite

**Figure 9:**
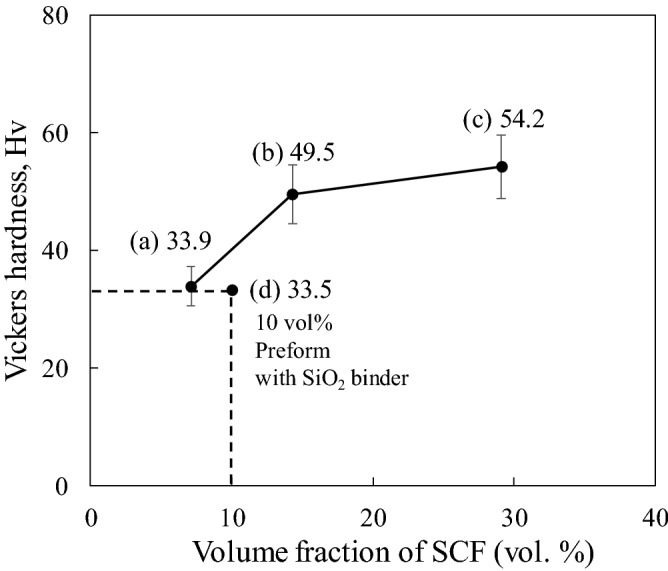
Vickers hardness of Cu-plated SCF/Al composites by different volume fractions of short carbon fiber.

The relative densities of fiber reinforced composite materials with CF 7.1 vol%, 14.3 vol% and 29.1 vol% were 2.63 kg/m^3^, 2.60 kg/m^3^ and 2.53 kg/m^3^, respectively. Figure 9 shows the Vickers hardness of Cu-plated SCF/Al matrix composites without preform manufacturing with different SCF volume fractions. The Vickers hardness of the A1070 matrix was 19.1 Hv. The Vickers hardness values of the Cu-plated SCF/Al matrix composites with 7.1, 14.3, and 29.1 vol% SCF was 33.9, 49.5, and 54.2 Hv, respectively, as shown in Fig. 9a–c.

The hardness of the composites containing SCFs increases with the increase in SCF content. All composites were harder than the A1070 matrix and dramatically improved by 76%, 161%, and 185%. The introduction of SCFs into the Al matrix produces fiber dispersion strengthening in the matrix. In addition, it was reported^11^ that there is an increase in hardness of the matrix surrounding SCFs due to the dissolving and diffusion of Cu from the surface of fibers, resulting in the solid solution strengthening in the composites.

**Figure 10:**
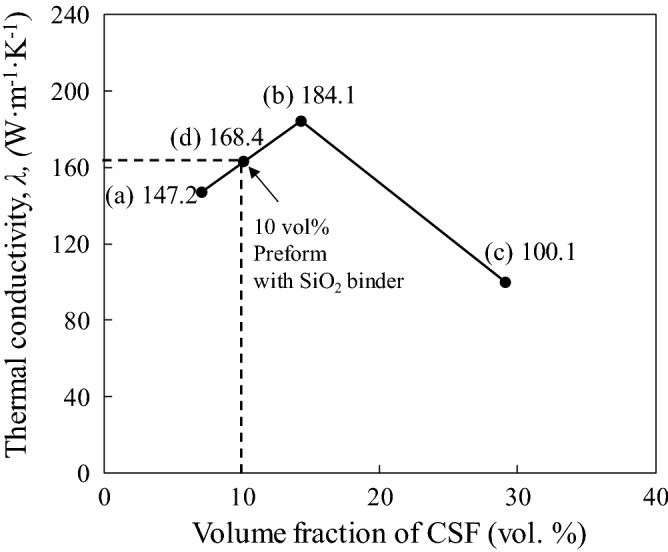
Thermal conductivity of Cu-plated SCF/Al composites by different volume fractions of short carbon fiber.

Figure 10 shows the thermal conductivity (TC) of the Cu-plated SCF/Al matrix composites without preform manufacturing with different SCF volume fractions, which were 147.2, 184.1, and 100.1 W m^−1^ K^−1^, respectively, as shown in Fig. 10a–c. It is seen that the TC first increases with the increase in SCF volume fraction. Cu-plated SCF/Al composite with 14.3 vol% SCFs, as seen in Fig. 10b, possessed the highest TC among the composites. The high TC was due to the excellent fiber dispersion in the composite, as shown in Fig. 5b. However, the composite containing 29.1 vol.% SCFs exhibited a drop in TC, as shown in Fig. 10c. According to the microstructure shown in Fig. 5c, SCFs agglomeration was observed due to the high volume fraction of fibers, and imperfect infiltration defects arose from the fiber clusters, causing a decrease in TC.

## Conclusions

A new fabrication process without preform manufacturing was developed for SCF/Al matrix composites. The significant results are summarized as follows:

1. A fairly uniform and continuous Cu layer is plated on SCFs via electroless plating. The fiber dispersion in the Cu-plated SCF/Al matrix composite is also improved, and the defects are minimized. The process can successfully produce composites with up to 29.1 vol% SCF without preform manufacturing. SCFs are uniformly distributed in the A1070 matrix with little agglomeration and showed better fiber dispersion than the composite fabricated with SiO_2_ binder.
2. The Hardness of composites containing SCFs increases with the increase in the SCF content. The hardness values of the composites containing 7.1, 14.3, and 29.1 vol% SCFs were 33.9, 49.5, and, 54.2 Hv, which are dramatic improvements of 76%, 161%, and 185%, respectively, over that of the A1070 matrix.
3. The thermal conductivity of composites containing SCFs increases first then decreases with the increase in the SCF content. The Cu-plated SCF/A1070 composite with 14.3 vol% SCF possessed the highest TC of 184.1 W m^−1^ K^−1^ among the composite, which was attributed to its excellent fiber dispersion without fiber clusters.

## Data Availability

The datasets generated and analysed during the current study are available from the corresponding author on reasonable request.
